# The mitochondrial‐derived peptide MOTS‐c: a player in exceptional longevity?

**DOI:** 10.1111/acel.12389

**Published:** 2015-08-20

**Authors:** Noriyuki Fuku, Helios Pareja‐Galeano, Hirofumi Zempo, Rafael Alis, Yasumichi Arai, Alejandro Lucia, Nobuyoshi Hirose

**Affiliations:** ^1^ Graduate School of Health and Sports ScienceJuntendo UniversityChibaJapan; ^2^ European University of MadridMadridSpain; ^3^ Research Institute of Hospital 12 de Octubre (‘i+12’)MadridSpain; ^4^ Research Institute ‘Dr. Viña Giner’, Molecular and Mitochondrial MedicineCatholic University of Valencia San Vicente MártirValenciaSpain; ^5^ School of MedicineCatholic University of Valencia San Vicente MártirValenciaSpain; ^6^ Center for Supercentenarian StudyKeio University School of MedicineTokyoJapan

**Keywords:** aging, centenarians, longevity gene, longevity regulation, mitochondria, mitochondrial DNA, mitochondrial DNA abnormalities, molecular biology of aging

## Abstract

Mitochondrial‐derived peptides (MDP) are encoded by functional short open reading frames in the mitochondrial DNA (mtDNA). These include *humanin*, and the recently discovered *mitochondrial open reading frame of the 12S rRNA‐c* (MOTS‐c). Although more research is needed, we suggest that the m.1382A>C polymorphism located in the MOTS‐c encoding mtDNA, which is specific for the Northeast Asian population, may be among the putative biological mechanisms explaining the high longevity of Japanese people.

## Background

The number of people aged ≥60 years is expected to almost triple by 2050, with the ‘*oldest old*’ group (>85 years) being the most rapidly expanding segment in Western societies (Waite, 2004). Among long‐lived individuals, those who reach exceptional longevity (EL, *i.e*., centenarians (≥100 years) and supercentenarians (SCs, ≥110 years)) are arguably the paradigm of successful aging (Andersen *et al*., 2012). Several genetic factors might contribute to EL, as suggested by the differences found in the frequency distribution of several genetic variants among centenarians compared with their ethnic‐matched referents of younger ages (Alexe *et al*., 2007; Ruiz *et al*., 2012; Garatachea *et al*., 2014). Factors related to inflammation (Basile *et al*., 2012), metabolism (Emanuele *et al*., 2014) or nutrition (Pareja‐Galeano *et al*., 2015), among others, can also influence the likelihood of reaching EL.

Japan has clearly the longest life expectancy in the world, as well as the highest number of SCs, as we recently reviewed (Santos‐Lozano *et al*., 2015). Thus, Japanese long‐lived people represent an interesting model to study the biology of EL, and to gain insight into the nature *vs*. nurture debate.

## Mitochondrial haplogroups and EL

Mitochondrial DNA (mtDNA) can influence EL (He *et al*., 2014). The 16,569‐bp human mtDNA contains 13 genes that codify proteins involved in mitochondrial oxidative phosphorylation (OXPHOS), as well as 2 rRNA and 22 tRNA genes that are necessary for protein synthesis within mitochondria (Mercer *et al*., 2011). Mitochondria are one of the most important players to understand the aging process at the cellular level as they are both the main source and target of oxidative damage (Gomez‐Cabrera *et al*., 2012). Mitochondrial dysfunction is in fact a main hallmark of aging, which is partly caused by accumulation of mtDNA damage as we age (Lopez‐Otin *et al*., 2013). Thus, because mtDNA haplotypes or haplogroups (*i.e*., characteristic clusters of tightly linked mtDNA polymorphims that form continent‐specific genotypes) might influence individual susceptibility to mtDNA damage, they could also influence EL in a continent‐ or ethnic‐specific manner (Pinos *et al*., 2012). For instance, the association between mtDNA and EL is controversial in Spanish people, with Pinos *et al*. reporting no association between mtDNA haplogroups and EL (Pinos *et al*., 2012) but Dominguez‐Garrido and co‐workers finding that the Caucasian haplogroup J (which would be associated with lower mtDNA damage) might confer a higher chance to attain high longevity (85+years) compared with other haplogroups in Northern Spaniards (Domínguez‐Garrido *et al*., 2009). On the other hand, although mtDNA haplogroups D4b2b, D4a, and D5 are not associated with type 2 diabetes (Fuku *et al*., 2007), they are linked with EL in Japanese population (Alexe *et al*., 2007; Bilal *et al*., 2008). We also showed that the mtDNA m.1382A>C polymorphism, which is specific for the ancestor haplogroup D4b2, is associated with EL in the Japanese population (Alexe *et al*., 2007).

## Mitochondrial‐derived peptide MOTS‐c

Mitochondrial‐derived peptides (MDP) are encoded by functional short open reading frames in the mtDNA. These include *humanin*, a 24‐amino acid peptide encoded in the 16S rRNA region with strong cytoprotective actions (Hashimoto *et al*., 2001) and the recently discovered *mitochondrial open reading frame of the 12S rRNA‐c* (MOTS‐c), which is a 16‐amino acid peptide that regulates insulin sensitivity and metabolic homeostasis (Lee *et al*., 2015). We have recently suggested that MOTS‐c might also be involved in the aging process (Alis *et al*., 2015).

**Figure 1:**
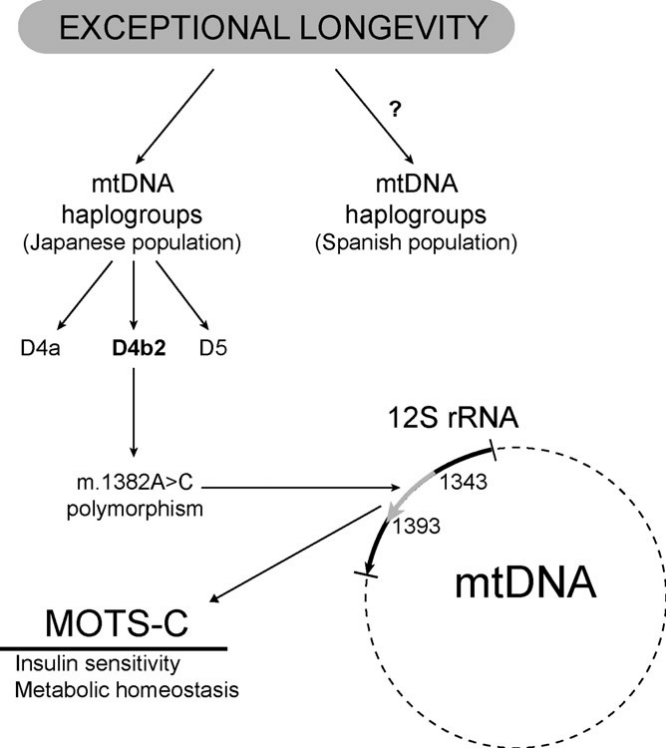
Putative biological link between the novel mitochondrial‐derived peptide MOTS‐c and exceptional longevity through the m.1382A>C mtDNA polymorphism. See text for abbreviations.

The aforementioned m.1382A>C polymorphism is located in the MOTS‐c encoding mtDNA, that is a 51‐bp short open reading frame in the 12S rRNA region, from positions m.1343 to m.1393 (Table 1). The m.1382A>C variation causes a Lys14Gln replacement in the MOTS‐c peptide equivalent to nucleotide position 1382 of the mtDNA; this is likely to have functional consequences, as the physicochemical difference between the original and the altered aminoacid residues is relatively high, with a Grantham value of 53, that is, above the average value (=50) that differentiates radical from conservative single amino acid replacements (Grantham, 1974). This amino acid replacement is also predicted to have a functional effect with the PROVEAN (PROtein Variation Effect ANalyzer) tool (http://provean.jcvi.org), that is, yielding a score of −4.000, below the specifically predicted cutoff score (=−2.5) above which the variant would be ‘neutral’ (Choi *et al*., 2012). The m.1382A>C polymorphism is specific for the Northeast Asian population and may be among the putative biological mechanisms explaining the high longevity of Japanese people. Further, MOTS‐c is an important ‘mitokine’, with this term referring to mitochondrial‐derived signals that impact other cells in an endocrine‐like manner (Fig. 1).

**Table 1:** The m.1382A>C polymorphism in the mtDNA region 12S rRNA (highlig‐hted in bold) causes Lys14Gln replacement in the mitochondrial‐derived peptide MOTS‐c

Nucleotide position^a^	Nucleotide sequence	Aminoacid (3‐letter code)	Aminoacid position
1343	atg	Met	1
1346	agg	Arg	2
1349	tgg	Trp	3
1352	caa	Gln	4
1355	gaa	Glu	5
1358	atg	Met	6
1361	ggc	Gly	7
1364	tac	Tyr	8
1367	att	Ile	9
1370	ttc	Phe	10
1373	tac	Tyr	11
1376	ccc	Pro	12
1379	aga	Arg	13
**1382**	**[A>C]aa**	**Lys>Gln**	**14**
1385	cta	Leu	15
1388	cga	Arg	16
1391	tag	Stop	

a Position number based on the entire mtDNA sequence.

## Conclusions

We suggest a biological link between MOTS‐c and extended lifespan through the putative endocrine action of this mitokine. Further mechanistic research is needed to determine the functional significance of the m.1382A>C polymorphism and the potential influence of MOTS‐c in the human aging process.

## Funding

This work was supported in part by grants from the programs Grant‐in‐Aid for Scientific Research (B) (15H03081 to N.F.) from the Ministry of Education, Culture, Sports, Science and Technology of Japan. Research in the field by A.L. is supported by Fondo de Investigaciones Sanitarias (FIS, grant#PI12/00914) and Fondos FEDER.

## Author′s contributions

All authors have: (i) made substantial contributions to conception and design (NF, HP‐G, HZ, RA, AL, NH, YA); (ii) drafted the article (NF, HP‐G, HZ, RA) or revised it for critically for important intellectual content (AL, NH, YA); and (iii) gave final approval of the version to be published (NF, AL, NH, YA).

## Conflict of interest

None declared.
